# Rumination, mood, and maladaptive eating behaviors in overweight and healthy populations

**DOI:** 10.1007/s40519-020-00857-z

**Published:** 2020-02-18

**Authors:** Monika Kornacka, Kamila Czepczor-Bernat, Piotr Napieralski, Anna Brytek-Matera

**Affiliations:** 1 Institute of Psychology, SWPS University of Social Sciences and Humanities, ul. Chodakowska 18/31, 03-815 Warsaw, Poland; 2 PSITEC EA 4072, University of Lille, Villeneuve d’Ascq, France; 3 Institute of Psychology, University of Wroclaw, Wroclaw, Poland; 4 Institute of Information Technology, Lodz University of Technology, Lodz, Poland

**Keywords:** Rumination, Emotional eating, Uncontrolled eating, Overweight, Ecological momentary assessment

## Abstract

**Purpose:**

The literature suggests that rumination (i.e., repetitive thinking about 1 or more negative concerns that is perceived as difficult to control) is linked to impaired emotional regulation and increases negative affect. Not only individuals suffering from overweight or obesity, but also healthy individuals might use emotional eating as a coping strategy to deal with negative affect caused by rumination. The aim of the present study was to determine the link between rumination and maladaptive eating strategies in participants with normal weight and overweight/obesity using trait and ecological momentary measures.

**Method:**

In Study 1, 88 individuals from overweight/obese (*N* = 33) and control group (*N* = 50) filled in a series of questionnaires assessing trait rumination, and eating behaviors. In Study 2 momentary affect, rumination and eating behavior of 26 participants were assessed using ecological momentary assessment (EMA) methodology.

**Results:**

In Study 1, the moderated mediation model revealed that emotional eating mediates the link between rumination and uncontrolled eating or snacking, but only in healthy participants and not in the participants with overweight. The results of Study 2 suggest that when both momentary rumination and sad mood are entered into the model predicting momentary daily emotional eating, only rumination remains a significant predictor of emotional eating. This relationship is not modified by the fact that the participants are from healthy controls or the overweight/obese group.

**Discussion:**

Study 1 provided evidence on the differential role of emotional eating in participants with normal weight and with overweight. Study 2 provided initial insights into the role of momentary mood and momentary repetitive thinking in the use of emotional eating in participants’ everyday lives. The differences in group effect in trait and EMA measures indicated also the importance of considering the consciousness of using rumination and emotional eating, while studying those processes in individuals with overweight.

**Level of evidence:**

Level III, case-control analytic study.

## Introduction

The category of abnormal eating behavior is relatively extensive [1]. On the one hand, research and clinical observations on eating behavior suggest that dietary abnormalities are common in individuals with excessive body weight [2–5]. In this group, food might be considered as a strategy of coping with stress and negative emotions [6]. On the other hand, dietary abnormalities (i.e., individual’s dietary intake which does not contain the right amount of nutrients for healthy functioning) may be occasional and may also occur in individuals with normal body weight [7]. The aim of the present study was to determine how cognitive processes, i.e., rumination, might affect abnormal eating behaviors in the context of emotional regulation in participants with overweight/obesity and in healthy participants. The objective was not only to explore this relationship at the trait level (Study 1), but also to provide preliminary results on the link between those variables in participants’ everyday lives using ecological momentary assessment (EMA, Study 2).

The literature provides a large perspective on the link between emotional regulation and maladaptive eating behaviors, explaining why people tend to use eating as emotional regulation strategy and how maladaptive eating behaviors became a habitual response in the context of distress [8]. The current literature brings some first evidence for the hypothesis suggesting that the use of eating as maladaptive emotional regulation strategy might be determined by the negative reinforcement, i.e., eating is decreasing negative affect [8–10]. Leehr and collaborators [9], after examining 18 experimental studies on the link between eating and emotional regulation in obese and binge eating patients, concluded that emotional relief after eating might be one of the factors maintaining maladaptive eating behaviors. However, they underline also that the experimental evidence is too small to definitely confirm this hypothesis. Thus, it seems crucial to further determine what are the cognitive or behavioral processes underlying the link between eating behaviors and emotional regulation. The literature suggests that one of the cognitive processes leading to maladaptive eating behavior might be ruminative thinking [11–14]. Rumination refers to repetitive dwelling on one more negative issues that is perceived as difficult to control [15]. High ruminators, to deal with negative affect caused by repetitive negative thinking, might use eating as an emotional regulation strategy, and, consequently this strategy, as suggested by Berg and collaborators [8], might become a habit, particularly in a distressing situation. The role of disordered eating behavior as a maladaptive attempt of regulating emotions elicited by rumination seems to be in line with the results of two recent meta-analysis confirming, that rumination is generally associated with abnormal eating and that this relation is stronger in individuals suffering from eating disorders, particularly anorexia nervosa and bulimia [14, 16]. This link is also observed among patients with obesity, in whom rumination is associated with the severity of eating disorders even after accounting for over-evaluation of shape/weight [14, 17]. Additionally, Smith and colleagues [14], in their meta-analysis, suggest that the causal relation between rumination and disordered eating might be reciprocal. Despite the growing amount of research on rumination in eating- and weight-related disorders, the interplay between rumination, negative affect regulation, and maladaptive eating behaviors remains unclear. Thus, testing this relation requires a closer look at the literature linking abnormal eating behaviors to both, emotional regulation and rumination. As Smith et al. [14] underline, in the conclusion of their meta-analysis on the link between eating disorders and rumination, it is necessary to include rumination in the model of eating disorders but also to explore the potential mechanism of its functioning in the context of abnormal eating behaviors.

While linking abnormal eating behavior to affect, particularly in the context of overweight or obesity, it is necessary to focus not only on cognitive restrictions but also on uncontrolled and emotional eating [9, 17–21]. Uncontrolled eating is defined as the consumption of excessive amounts of food with a sense of a lack of control over food [21, 22]. Based on numerous studies, it can be concluded that excessive consumption of food linked to uncontrolled eating leads to weight gain and becoming overweight or obese [9, 23]. Uncontrolled eating is also linked to snacking, which is defined as repeated eating between meals [1, 24]. Previous studies have shown that snacking is most often associated with eating sweets or fast food, and this has an impact on weight gain and on the effects of the treatment of excessive body weight [4, 25]. One’s feeling of losing control over the eaten amount of food and eating in response to emotional distress, called emotional eating, are two different types of maladaptive eating behaviors; they can, however, mutually affect each-other [6, 21].

In the literature, there is no convincing evidence for the causal relationship between affect and eating disorder behavior like emotional or uncontrolled eating. On the one hand, we can hypothesize that negative affect increases over time until the point at which maladaptive eating behavior occurs as an emotional regulation strategy [26]. Previous research has shown that momentary affective states are associated with overeating and binge eating episodes in individuals with obesity [10]. On the other hand, the empirical evidence of the impact of maladaptive eating behaviors on negative affect remains inconsistent. Some research suggests that negative affect decreases after maladaptive eating behaviors [27]; while, other studies suggest that it does not decrease or continues to increase [28]. This inconsistency seems to be particularly important in the context of disordered eating behavior becoming a habit because of the attempts to regulate negative emotions.

When considering negative affectivity in the context of eating behaviors, it is also necessary to take into account a situation where one has a low consciousness of experienced emotions, and thus, feelings might be confused with signals of physiological hunger. According to the literature, this phenomenon increases the risk of emotional regulation by food [29]. This assumption was previously suggested by the classic psychosomatic theory [30]. Food acts as a negative emotion regulator and the process of overeating is also caused by the interpretation of emotional experiences as hunger [30, 31]. Further, the current mindfulness-based eating solution argues that individuals with overweight and obesity consume food, ignoring physical body signals while eating under the influence of emotions [7, 30, 32, 33]. Among overweight and obese women, emotional eating is positively associated with both emotion dysregulation and negative affect; however, mindful eating does not buffer the impact of emotional dysregulation and negative affect on emotional eating [34]. Those results suggest that food loses its original energy function and serves to satisfy emotional comfort.

The recent literature, including two meta-analyses [14, 16], suggest that the complexity of the link between affect and disordered eating behaviors requires taking into account also cognitive processes (i.e., rumination) involved in this link. Rumination might be considered as a process moderating the relationship between negative affect and emotional regulation by food [17–20, 35]. Rumination, i.e., difficult to control repetitive negative thinking [15], disrupts cognitive functioning and is associated with emotional dysregulation and higher negative affect [36]. It has been found to be involved in the maintenance of several types of maladaptive strategies focused on dealing with stress, including eating [14, 37]. On the one hand, recent studies have suggested that the repetition of specific thoughts related to body and body mass index can become a source of compensatory behaviors such as binging, purging, and exercising [38]. On the other hand rumination itself might increase the amount of body-related negative cognitions [14].

The potential impact of rumination on maladaptive eating behaviors might be operating at several levels. First, both rumination and disordered eating behaviors are linked to self-control failures [39, 40]. Rumination consumes a significant part of our observation and self-control resources, which may result in higher food-related unawareness [7, 39]. Additionally, Watkins suggested that rumination is linked to dysregulation  in the level of goal and action identification that might cause the aforementioned lower emotional awareness [41] and difficulties in negative affect regulation [42, 43]. Moreover, Laghi et al. [44] observed that poor emotional awareness might be also linked to the need for controlling thoughts—one of the main consequences of rumination. This need for control over one’s thoughts might play the role of a moderator between lack of emotional awareness and eating abnormalities (binge eating) according to this recent study [44]. The results of Laghi et al.’s [44] study underline the necessity of focusing on a particular feature of ruminative thinking linking this process to self-control resources and suggesting that rumination might cause a depletion of those resources and enhance the use of automatic, habitual emotional regulation strategies [45].

Another mechanism by which rumination might link emotional regulation to eating behavior is actual–ideal self-discrepancy [46]. Maladaptive eating behaviors and transgression of the eating rules that given individual belief she/he should follow create a discrepancy between reality and desired behavior. This discrepancy might be particularly salient when the individual is preoccupied with food and uses eating as a strategy of emotion regulation. According to Martin and Tesser [47], this kind of discrepancy triggers ruminative thinking. One’s engagement in repetitive thinking might cause a long-term increase in negative affect [42] and consequently enclose this person in a kind of vicious circle where rumination causes negative affect further regulated by eating behaviors, causing, in turn, guilt and consequently more ruminative thinking.

The aim of the present series of studies was first to determine the link between the general tendency to ruminate (trait level and a particular rumination feature, mental capacities captured by rumination linking this cognitive process to self-control failure) and maladaptive eating strategies (as defined above: emotional eating, uncontrolled eating, and cognitive restraint) in participants with normal weight and with overweight/obesity (Study 1). Based on the current literature, we hypothesized that emotional eating might play a mediator role between maladaptive features of rumination (i.e., lack of control over repetitive thinking and reduced cognitive resources due to rumination) and uncontrolled eating behavior, particularly in the group of participants with overweight or obesity.

The second aim of the present study was to explore the interplay between rumination, maladaptive eating behaviors, and mood using daily sampling to give first insights into the role of momentary mood and repetitive thinking in the use of emotional eating in participants’ everyday lives (Study 2). According to the literature, both momentary affect and rumination should predict emotional eating in participants’ daily life; this relation should be stronger in participants with overweight or obesity.

## Study 1

### Methods

#### Recruitment

Participants were recruited via an on-line advertisement on Facebook. The recruitment took place in January and February 2017. The inclusion criteria were (1) age (adulthood): 18–65 years old, (2) BMI: normal body weight—18.5–24.9 kg/m^2^ (control group) or overweight ≥ 25 kg/m^2^ (overweight group), (3) without diagnosis and co-occurrence of eating disorders (based on participant’s declaration). In the advertisement, participants were invited to take part in a study on the link between cognitive processes and eating behaviors.

#### Participants and procedure

Ninety participants completed a series of online questionnaires. The data from 2 participants were removed due to missing information. The final sample was composed of 50 participants from a normal weight group and 38 participants from an overweight group. All the participants agreed to participate in the study after reading and on-line consent form and choosing between mentions “I do agree to participate” and “I don’t agree to participate”. Participants did not receive any compensation for their participation in the study. Prior to their participation, the participants read an online information letter and agreed to take part in the study.

#### Measures

*The Perseverative Thinking Questionnaire* [48, 49]. The PTQ is a self-reported questionnaire assessing repetitive negative thinking (RNT) from a content-independent perspective. The questionnaire asked participants to describe how they typically thought about negative experiences or problems and to rate the extent to which each statement applied to them when they thought about negative experiences or problems using the 5-point Likert scale from 0 (never) to 4 (almost always). The questionnaire was composed of a single second-order factor, RNT, and three lower-order factors. The lower-order factors: core features of RNT (9 items) evaluating the main characteristics of repetitive negative thinking, unproductiveness of RNT (3 items) evaluating to what extent participants find the use of repetitive negative thinking unhelpful, and mental capacity captured by RNT (3 items) evaluating to what extent Repetitive negative thinking interferes with one’s current activities and is linked with the self-control failure. Cronbach’s alpha was of .95 for the total score and ranged from .64 to .92 for the subscales.

*The Three-Factor Eating Questionnaire (TFEQ-R18)* [21, 50]. The TFEQ-R18 measures 3 aspects of eating behavior: emotional eating (3 items), uncontrolled eating (9 items), and cognitive restraint (6 items). In the Polish study, Cronbach’s alpha reliability coefficient for “emotional eating” was .86, for “uncontrolled eating” was .84, and for “cognitive restraint” was .78.

*Eating and weight-related behaviors*. We also asked a series of questions assessing eating and weight-related behaviors. Participants reported on a 5-point Likert scale whether and how often they snacked during the day, what kind of snacks they chose, how many main meals they ate daily, and what kind of products they usually chose.

#### Data analysis

The data from Study 1 were analyzed first with correlation and second, with conditional process models. All the conditional process models were run using Process plug-in software for SPSS [51]. The number of bootstraps was set to 10,000. The lower-level confidence interval and the upper-level confidence interval for unconditional effects are presented in square brackets.

**Fig. 1:**
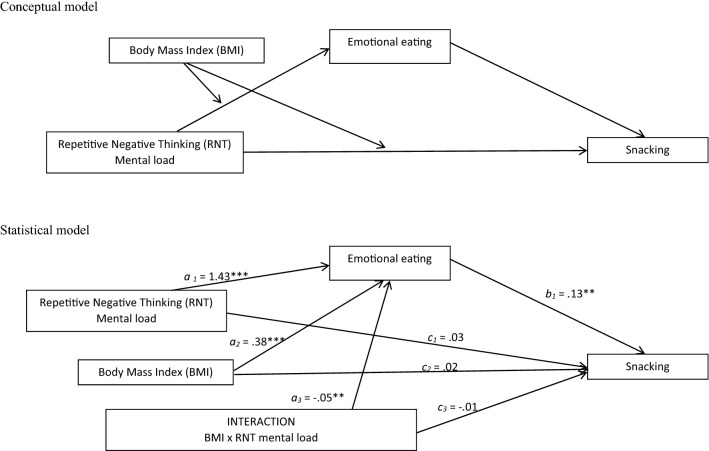
Conceptual and statistical model of moderated mediation the link between PTQ mental load and sneaking trough emotional eating, moderated by group status

**Fig. 2:**
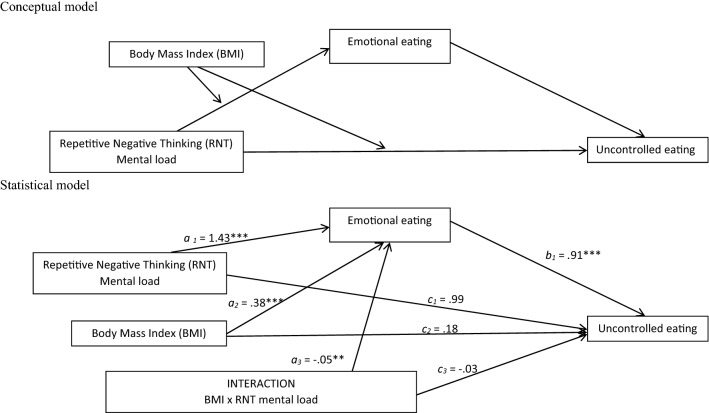
Conceptual and statistical model of moderated mediation the link between PTQ mental load and uncontrolled eating trough emotional eating, moderated by group status

To explore whether the link between repetitive negative thinking and uncontrolled eating or snacking can be mediated by emotional eating, we computed two models of moderated mediation (see Figs. 1 and 2).

### Results

#### Descriptive statistics and correlations between trait variables in overweight/obese and normal weight group

Descriptive statistics of demographic data are presented in Table 1. To explore how the general tendency to ruminate and eating behaviors evaluated as a trait variable are related in the normal weight and overweight groups, we conducted two sets of Pearson’s correlations (see Table 2).

**Table 1:** Descriptive statistics for normal weight and overweight groups

Variable	Normal weight (*N* = 50)		Overweight (BMI > 25) (*N* = 38)		Group comparison
	Mean	SD	Mean	SD	*T*(86)
Age	29.22	9.47	32.50	13.27	1.35
Weight (kg)	60.34	6.77	88.97	18.08	10.34***
Height (cm)	167.40	7.65	170.66	12.67	1.50
BMI	21.54	2.06	30.56	6.07	− 9.81***
PTQ total score	37.74	12.95	37.92	1.92	.06
PTQ core features	24.00	8.78	24.16	8.08	.09
PTQ unproductiveness	7.02	2.58	7.21	2.71	.35
PTQ mental load	6.72	2.48	6.55	3.40	.79
TFEQ uncontrolled eating	8.68	5.07	9.76	5.81	.93
TFEQ emotional eating	3.08	2.62	3.32	2.95	.37
TFEQ cognitive restrictions	9.64	3.80	10.21	4.05	.68

*BMI* body mass index, *PTQ* Perseverative Thinking Questionnaire, *TFEQ* Three-Factor Eating Questionnaire

***p < .001

**Table 2:** Pearson’s correlations between trait variables in normal weight group (lower part of the table) and overweight group (upper part of the table)

Variable	1.	2.	3.	4.	5.	6.	7.	8.	9.
1. PTQ total score	–	.96***	.79***	.89***	− .07	− .12	.15	− .31	− .13
2. PTQ core features	.98***	–	.62***	.77***	− .16	− .21	.26	− .30	− .01
3. PTQ unproductiveness	.86***	.77***	–	.73***	.15	.08	− .05	− .24	− .21
4. PTQ mental load	.85***	.77***	.70***	–	− .01	− .01	− .01	− .29	− .32
5. TFEQ uncontrolled eating	.51***	.46**	.52***	.53***	–	.54***	− .15	.10	− .42**
6. TFEQ emotional eating	.39**	.33*	.38**	.48***	.52***	–	− .16	.41*	− .17
7. TFEQ cognitive restrictions	.35*	.40**	.26	.13	.21	− .07	–	− .13	− .08
8. BMI	− .09	− .05	− .05	− .21	− .03	.10	.28	–	.29
9. Age	− .37***	− .33*	− .37**	− .37*	− .41**	− .23	− .21	.08*	–

*PTQ* Perseverative Thinking Questionnaire, *TFEQ* Three-Factor Eating Questionnaire

*p < .05

**p < .01

***p < .001

There are some crucial differences between the correlations in the two groups. Rumination was related to eating behaviors in the normal weight group but not in the overweight group. In the normal weight group, the PTQ general score was correlated with uncontrolled eating, emotional eating, and cognitive restraint, this correlation was not significant in the overweight group. The PTQ general score and emotional or uncontrolled eating from the TFEQ-R18 correlations were significantly different in the two groups, respectively *z* = 2.87, *p* < .01; *z* = 2.41, *p* < .01. However, the PTQ general score and the TFEQ-R18 cognitive restraint correlation were not significantly different in the two groups (*z* = .95, ns).

#### Moderated mediation models

The results suggest that the model of moderated mediation of the link between the PTQ mental load and snacking through emotional eating, moderated by BMI was significant *R* = .46; *F*(4,84) = 7.30; *p *< .001; MSE= 6.22 (see Fig. 1). The index of moderated mediation (for emotional eating as a mediator and BMI as a moderator) was − .01; [− .03, − .001]. The conditional indirect effect of the PTQ mental load on snacking through emotional eating was significant in the individuals with lower BMI (− 1SD) (*B *= .07; [.02, .13]) and mean BMI (*B *= .05; [.01, .08]) but was not significant in the individuals with high BMI (+ 1SD) (*B *= .007; [− .03, .04]. The results of the conditional direct effect suggest that the PTQ mental load does not directly affect snacking independently of BMI.

Similar results were obtained for the model of moderated mediation of the link between the PTQ mental load and uncontrolled eating through emotional eating, moderated by BMI, *R* = .55; *F*(4,83) = 8.98; *p *< .001; MSE= 21.32 (see Fig. 2). The index of moderated mediation (for emotional eating as a mediator and BMI as moderator) was − .04; [− .07, − .01]. The conditional indirect effect of the PTQ mental load on uncontrolled eating through emotional eating was significant in the individuals with lower BMI (− 1SD) (*B *= .50; [.21, .84]) and mean BMI (*B *= .31; [.11, .56]) but was not significant in the individuals with high BMI (+ 1SD) (*B *= .04; [− .19, .27]. The results of the conditional direct effect suggest that the PTQ mental load does not directly affect uncontrolled eating independent of the BMI.

### Discussion

According to our predictions, results at the trait level showed that rumination, and more precisely its particular feature, i.e., cognitive resources captured by rumination, is linked to uncontrolled eating and that this relationship was mediated by emotional eating. Although the collected data have cross-sectional character, it corroborates the theoretical perspective on rumination increasing uncontrolled eating because individuals try to regulate their negative affect via food intake in line with the results of previous experimental studies [9] and recent meta-analyses [14, 16]. However, contrary to our hypothesis on this relation in healthy individuals and participants with overweight or obesity, this mediation model was significant only for individuals with lower BMI. In the overweight group, there was no direct or indirect effect of rumination on uncontrolled eating. The results might be explained by the fact that in individuals with overweight and obesity, uncontrolled eating became a habitual emotional regulation strategy. A recent study underlines the differences in emotional regulation in individuals with high, habitual use of rumination comparing to those for whom rumination is not a preferential emotional regulation strategy [52]. Watkins and Nolen-Hoeksema [45] suggested that executive resources are no longer involved in the link between rumination and depression once rumination becomes a habitual, automatic emotional regulation strategy. Thus, a similar mechanism might be postulated in individuals with overweight. When an individual experiences negative affect, he/she automatically uses maladaptive eating behaviors to regulate it, and consequently the cognitive resources captured by rumination or emotional eating are no longer involved in this relationship, contrary to the mechanism in the healthy population. This result raises an important question about the role of rumination-focused intervention in maladaptive eating behaviors. If we follow the hypothesis that resources captured by rumination and emotional eating are involved in maladaptive eating behavior only before it becomes a habitual, automatic response, it might suggest that rumination is a good target for maladaptive eating behavior prevention, but not for the treatment of a fully developed eating or weight-related disorder.

In the perspective of differences between healthy participants and individuals with overweight/obesity in terms of automatic vs. controlled use of eating as an emotional regulation strategy, it is interesting to underline the role self-control and executive resources in the link between rumination and eating. In healthy individuals, the process of eating itself might be a source of rumination [7]. By categorizing food into a “forbidden” or “right” category, a diet might be defined as “healthy”, maintaining a “healthy” diet is often one’s goal and can be considered in terms of success or failure. Following this goal is linked to food restriction that requires and might exhaust cognitive resources [53]. In the event of severe stress or negative affect often caused by rumination, food restrictions are broken due to the lack of self-control resources already captured by rumination [7, 53], which leads to the consumption of forbidden food, the feeling of guilt [7] and a discrepancy between the ideal and actual self. This discrepancy might restart ruminative thinking [47]. Schlinkert and Koole [54] confirmed the role of inhibitory control resources in the link between rumination and eating behaviors. Moreover, Wang and Borders [35] suggested that also angry rumination might be itself depleting, which might accentuate this effect of losing control of food intake. In this vicious circle, anxiety about gaining weight strengthens a low sense of efficacy and might become an additional source of rumination. Finally, it is worth pointing out that rumination is a form of escape from the here and now. However, instead of reducing the level of stress, it has an opposite effect and leads to an increase of negative affect [43, 55] and creates a need for emotional regulation leading often to maladaptive eating behaviors [7].

Furthermore, according to the previous studies and in a perspective of self-control role in the process, when planning a change in dietary behavior, in addition to emotional eating and ruminating [56], it is also needed to take into account snacking in the group of individuals with different body weight [1, 23, 56]. Snacking is considered as a chronic, objectively maladaptive eating behavior, but individuals often do not assess it as abnormal or relevant and consequently it is difficult to detect and change [1, 23, 56]. According to the results of our study, rumination might be a potential trigger in addressing snacking or uncontrolled eating in eating- and weight-disorder prevention. It is, however, necessary to underline, that in the present study, snacking was evaluated by a single-item self-reported question. Although several studies showed that one-item questions correlate highly with self-reported behaviors evaluated by scale [57–59], it would be interesting, in the further research, to evaluate this behavior through more ecological measures.

## Study 2

### Methods

#### Recruitment

37 participants from Study 1 volunteered for participation in Study 2. They were given a login and a password to activate the application on their mobile phone.

#### Participants and procedure

Among 37 participants, 11 did not activate the applications or retrieved from the study during the data collection resulting in a final sample of 26 participants. All the participants agreed to participate in the study after reading and on-line consent form and choosing between mentions “I do agree to participate” and “I don’t agree to participate”. Participants did not receive any compensation for their participation in the study. Descriptive data for the participants of Study 2 are presented in Table 3.

**Table 3:** Descriptive statistics for EMA sample

Variables	Normal weight (*N* = 11)			Overweight (BMI > 25) (*N *= 15)			Group comparison
	Mean	SD	Comparison with trait sample	Mean	SD	Comparison with trait sample	*t*(86)
Age	24.18	8.75	2.06*	27.40	9.14	1.98	.90
Weight	58.72	6.46	.89	86.67	18.83	.63	4.69***
Height	171.53	14.81	1.37	164.64	7.36	.34	1.41
BMI	21.66	2.00	.59	29.29	2.27	1.05	4.53***
PTQ total score	40.36	15.76	.76	42.93	10.49	2.00*	.50
PTQ core features	25.00	10.49	.42	26.53	5.96	1.48*	.47
PTQ unproductiveness	7.27	2.57	.36	8.33	2.53	2.16*	1.05
PTQ mental load	8.09	3.24	2.15*	8.07	3.08	2.35*	− .19
TFEQ uncontrolled eating	8.82	5.07	.10	10.93	4.64	1.00	1.08
TFEQ emotional eating	3.83	2.48	1.06	3.40	1.95	.14	− .48
TFEQ cognitive restrictions	9.73	2.68	.08	10.53	3.44	.39	.64

*BMI* body mass index, *PTQ* Perseverative Thinking Questionnaire, *TFEQ* Three-Factor Eating Questionnaire

*p < .05

***p < .001

Prior to their participation, the participants read an online information letter and agreed to take part in the study. After completing the questionnaires, the participants who agreed to also take part in the EMA study downloaded a mobile application on their phones. The application was developed purposefully for use in the present study and operated on the Android system. It was developed as a part of Polonez 2 research project directed by the first author in the cooperation with the Technical University of Lodz, Poland. The application was directly linked to an on-line platform collecting the data. Participants were instructed that the application would send random auditory signals and “time for assessment” information five times a day for 5 days. The signals were sent randomly once in five 2-h intervals between 8 a.m. and 6 p.m. The participants had 15 min to answer a series of questions (see “Measures” section).

#### Measures

*Momentary emotional eating evaluation*. To assess daily emotional eating, we adapted the Emotional Eating subscale from the TFEQ-18. It consisted of 3 TFEQ statements evaluated on a 7-point Likert scale in answer to “Last time I ate because…” (1) “I was feeling sad”; (2) “I was consolating myself”; (3) “I was feeling nervous”; the additional statement “I was hungry” was added. The Cronbah’s Alpha was of .84 for the EMA.

*The brief state rumination inventory* [55, 60] is a short self-reported scale developed to assess the state of ruminative self-focus. It is composed of a single dimension of 4 items (“Right now, I wonder why I react the way I do”; “ Right now, I am conscious of my feelings”—inversed coding item; “Right now I am thinking how sad I feel”; “Right now, I am thinking what is the meaning of my inner feelings”) evaluated on a 7-point Likert scale. Cronbah’s Alpha for this scale in EMA was of .72.

*State mood evaluation* [61]. The mood rate is based on two questions assessing emotional state. Participants are asked to answer the question “Right now I am feeling…” by choosing a point on two bipolar six-points scales (content–discontent and well–unwell).

#### Checklist for reporting EMA studies (CREMAS [62])

1. *Sampling and measures* 37 participants using an application downloaded to their personal smartphone (with Android operating system).
2. *Schedule* Data were collected in one wave, during 5 days, with 5 signals a day.
3. *Technology and administration* use of the platform linked to the application created purposefully for the study.
4. *Prompting strategy* random interval contingent.
5. *Response and compliance* latency—15 min; attrition—11 participants dropped out from the study; compliance rate—38% of prompted questions.

#### Data analysis

Data in Study 2 were analyzed using hierarchical linear modeling (HLM 7.0; [63]) to accommodate the multilevel structure of the repeated assessments in daily life and to model between-person differences in mood–emotional eating and rumination–emotional eating relationships.

### Results

#### Descriptive statistics

In the momentary measures, we obtained a total of *N* = 256 valid scores at level 1 nested within 26 participants (*M *= 8.69, SD= 5.93). No significant differences between individuals with normal weight and overweight/obesity in the frequency of responses were observed (*t*(24) = − .50, *M *= 8, SD = 5.76 for normal weight, *M *= 9.2, SD = 6.20 for overweight). The frequency was not related to any trait measures (PTQ, TFEQ-R18, or self-reported body satisfaction). Descriptive statistics on trait variables and group comparison with the trait analysis sample are presented in Table 3.

The participants who volunteered to take part in Study 2 had statistically higher mental loads due to rumination in both the normal weight and overweight group. Additionally, those who were in the overweight group and volunteered for the Study 2 had significantly higher general PTQ scores, core features, and unproductiveness compared to the individuals with overweight who did not volunteer (see Table 4).

**Table 4:** Descriptive statistics for momentary measures

Measure	Mean	SD	Variance	
			Moment-level 1	Person-level 2
Emotional eating	5.64	3.52	7.42	5.04
Depressed mood	2.72	1.73	2.17	.72
Anxiety	2.48	1.71	2.27	.63
Rumination	22.20	6.98	27.81	20.81

#### Momentary mood and emotional eating

First, in the null models without any predictors, we estimated the mean, standard deviation and variance component at within- (observation) and between-person (person) level of analysis for each variable, see the model (1) below and Table 4.

1 $$\begin{aligned} & {\text{Observation-level}}\; \left( {\text{level 1}} \right){y_{ij}} = {\beta_{0j}} + {r_{ij}} \\ & {\text{Person-level}}\left( {\text{level 2}} \right){\beta_{0j}} = {\gamma_{00}} + \, {u_{0j}}. \end{aligned}$$

Further, momentary emotional eating was included in the model as the outcome and self-reported momentary sad mood as level 1 predictor, see the model (2) below.

2 $$\begin{aligned} & {\text{Observation-level}}\; \left( {\text{level 1}} \right) \; {{\text{Emotional eating}}_{ij}} = {\beta_{0j}} + {\beta_{1j}}*\left( {{{\text{Sad mood}}_{ij}}} \right) + {r_{ij}} \\ & {\begin{aligned} {\text{Person-level}}\; \left( {\text{level 2}} \right) \; {\beta_{0j}} & = {\gamma_{00}} + \, {u_{0j}} \\ {\beta_{1j}} & = {\gamma_{10}} + {u_{1j}}. \end{aligned}} \end{aligned}$$

The results suggest that self-reported sad mood was a significant predictor of emotional eating (see Table 5). To explore the impact of participants’ BMI on the relation between momentary mood and emotional eating, we included this variable in the model at level 2 (see model 3 below). This relationship was not affected by participants’ BMI (*γ *=− .02, *t* = − 1.64, *p *= .11).

3 $$\begin{aligned} & {\text{Observation-level}}\left( {\text{level 1}} \right)\; {{\text{Emotional eating}}_{ij}} = {\beta_{0j}} + {\beta_{1j}}*\left({{{\text{Sad mood}}_{ij}}} \right) + {r_{ij}} \\ & {\begin{aligned} {\text{Person-level}} \; \left( {\text{level 2}} \right){\beta_{0j}} & = {\gamma_{00}} + {\gamma_{01}}*\left( {BM{I_j}} \right) + {u_{0j}} \\ {\beta_{1j}} & = {\gamma_{10}} + {\gamma_{11}}*\left( {{\text{BM}}{{\text{I}}_j}} \right) + {u_{1j}}. \end{aligned}} \end{aligned}$$

**Table 5:** Relationship between mood, repetitive thinking and emotional eating (outcome) in everyday life

	Emotional eating		
	Coefficient	*t*	*p*
Depressed mood	.23	2.27	.03
Anxiety	− .08	.75	.46
Rumination	.10	2.06	.05

Mood states are based on assessments made at the same assessment period to the assessment of rumination and emotional eating. All mood states were group-mean centered, and therefore reflect deviation from an individual’s average level of depressed, anxious mood or repetitive thinking

Momentary ruminations was significant predictors of emotional eating; this effect was not modified by BMI (*γ * = − .01, *t* = − .58, *p * = .56). When both momentary rumination and sad mood were entered into the model predicting momentary emotional eating at level 1, only rumination remained a significant predictor (*γ * = .11, *t* = 2.41, *p * = .02 and *γ * = .17, *t* = 1.50, *p *= .14 for sad mood). At the trait level, the BMI did not affect this relationship (*γ * = − .03, *t* = − 1.85, *p * = .08 for sad mood slope and *γ * = − .01, *t* = − .14, *p * = .90 for momentary rumination slope).

### Discussion

The mediating role of emotional eating in the link between rumination and uncontrolled eating or snacking in participants with lower BMI explored in Study 1 suggested that momentary affect might play an important role in this relationship. Thus, we used ecological momentary assessment to preliminary test the impact of rumination and mood on emotional eating. Taking into account the previous studies [10, 64], we hypothesized that both momentary affect and rumination should predict emotional eating in participants’ daily life and that this relation should be stronger in participants with overweight or obesity. The results confirmed the role of rumination and affect on eating behaviors; however, this relation was not dependent on participants’ BMI. It is also important to underline, that when both predictors (momentary rumination and affect) are included in the model predicting emotional eating, only rumination remains a significant predictor, suggesting that in daily functioning, rumination might be further explored as a mediator of the link between affect and emotional eating as suggested by previous studies based on classical self-reported measures [64].

The general compliance in the study was low; it is, however, important to note that the participants did not receive any compensation for their participation. As indicated in the study of the feasibility of EMA procedures, financial compensation is crucial to maintain the participants’ compliance in this type of procedure [65]. Thus, this preliminary study on rumination and momentary eating behavior is subject to several limitations: the compliance in the study was low; consequently, the study is underpowered for the computation of cross-sectional analyses or lagged analyses. It was interesting to note that among participants from Study 1, those who volunteered for Study 2 had a significantly higher level of rumination, particularly on the dimension linked to cognitive resources captured by repetitive negative thinking. This fact underlines, on the one hand, the necessity of taking into account different aspects of rumination (e.g., abstract vs. concrete processing mode [43, 55]) in the further EMA studies, but, on the other hand, it might also suggest that participants suffering more from maladaptive rumination are more motivated to participate in more time-consuming studies on this subject. It would be interesting to explore, in further research, participants’ motivation (external vs. self-determined) to participate in the daily sampling studies and the impact of motivation on the compliance rate. Moreover, as the present study had a preliminary character and was, to our best knowledge, the first to explore rumination, mood and eating behavior in the ecological settings, the last signal was sent to the participants at 6 p.m.; thus, it is possible that an important part of maladaptive eating behaviors in the evening was omitted. This issue should be addressed in further studies.

Despite the small sample size and limitations mentioned above, it was important to disseminate the preliminary results of the study to give a further indication for the use of EMA in rumination and weight-related contexts. The results of Study 2 using daily sampling suggest that when both momentary rumination and sad mood are entered into the model predicting momentary emotional eating at level 1, only rumination remains a significant predictor of emotional eating. This relationship is not modified by the BMI level introduced at level 2 of analysis (*p* > .05). It is necessary to underline that an important limitation in the cross-level interaction in Stud 2 is the fact that they are underpowered due to the small sample size and low compliance. Thus, the lack of effect of BMI should be interpreted with precaution.

### General discussion

The present studies aimed at disambiguating the relationship between rumination, mood, and maladaptive eating behaviors (emotional eating, uncontrolled eating, and snacking) in individuals with normal weight and overweight/obesity. The first study explored, in a moderated mediation model, how emotional eating might affect the link between rumination and uncontrolled eating behavior. It seems that emotional eating might mediate this relation, but only in participants with lower or mean BMI, but not in those with high BMI. This moderating effect of BMI might suggest that in participants with higher BMI uncontrolled eating is an automatic, dominant response to psychological distress and other processes, like emotional eating or rumination, are no longer involved. Considering the results of the second study, where this moderating effect of BMI was not apparent, it seems crucial to discuss the potential implication of emotional consciousness. In the discussion of Study 1 the habit hypothesis [45] was presented as a potential explanation of lack of the involvement of emotional eating in the link between rumination and uncontrolled eating in participants with overweight. However, taking into account the results of Study 2, an alternative explanation might be put forward; i.e., individuals with overweight are not able to retrospectively report emotional eating. Previous research found that low interoceptive awareness is a possible risk factor for abnormal eating habits, for example, emotional overeating [66]. Additionally, the difficulties in retrospectively reporting the emotional context of eating behaviors in the overweight group might explain the differences between the trait and EMA results in our study.

In sum, the results of the two studies confirm the crucial role of ruminative thinking in the occurrence of emotional eating [64]. Study 1 underlines the role of rumination as a potential target of preventing disordered eating behaviors occurring occasionally, before they become a habitual emotional regulation strategy and might lead to overweight or obesity. However, the results of Study 2 brought some additional information suggesting that in both healthy and participants with overweight rumination might lead to increased emotional eating, even when controlling for negative affect. Those preliminary results of the momentary assessment study suggest that emotional eating might be not caused directly by negative affect but it is enhanced by repetitive dwelling on the negative issue and negative affect itself.

The results of two studies in line with the potential clinical implications underline a need for questioning retrospective self-reported measures and for further daily sampling studies in the population of individuals with overweight/obesity. It seems necessary to develop daily sampling methods adapted for eating-related problems and to introduce, in further research, more objective measures of momentary food intake or snacking that a simple self-report question that might address also the question of emotional awareness of participants.

Moreover, it seems necessary to continue to comparing daily sampling methods not only with traditional self-report measures but also with experimental studies in the laboratory settings, as the results of our studies suggest that, for example, the impact of participant’s BMI might be different dependent on used methodologies (EMA vs. classical self-report questionnaires). It would be also interesting to take into account other emotional regulation strategies linked to reduced emotional consciousness (e.g., expressive suppression) and the potential impact of comorbid psychological disorders (e.g., depression) that might be linked to the level and maladaptive feature of rumination [67].

#### What is already known on this subject?

Rumination is related to impaired emotional regulation and it might cause an increase in maladaptive eating. It is not clear what is the interplay between affect, rumination and impaired eating.

#### What does this study add?

The role of emotional eating in the link between rumination and uncontrolled eating is different in overweight vs. healthy individuals. Momentary rumination is linked to emotional eating.
